# A Personalized Spatial-Temporal Cold Pain Intensity Estimation Model Based on Facial Expression

**DOI:** 10.1109/JTEHM.2021.3116867

**Published:** 2021-09-30

**Authors:** Yikang Guo, Li Wang, Yan Xiao, Yingzi Lin

**Affiliations:** 1 Intelligent Human-Machine Systems LabMechanical and Industrial Engineering DepartmentCollege of Engineering, Northeastern University 27817 Boston MA 02115 USA; 2 College of Nursing and Health InnovationUniversity of Texas at Arlington 12329 Arlington TX 76019 USA

**Keywords:** Cold pain, facial expression, temporal information, personalized model

## Abstract

Objective: Pain assessment is of great importance in both clinical research and patient care. Facial expression analysis is becoming a key part of pain detection because it is convenient, automatic, and real-time. The aim of this study is to present a cold pain intensity estimation experiment, investigate the importance of the spatial-temporal information on facial expression based cold pain, and study the performance of the personalized model as well as the generalized model. Methods: A cold pain experiment was carried out and facial expressions from 29 subjects were extracted. Three different architectures (Inception V3, VGG-LSTM, and Convolutional LSTM) were used to estimate three intensities of cold pain: No pain, Moderate pain, and Severe Pain. Architectures with Sequential information were compared with single-frame architecture, showing the importance of spatial-temporal information on pain estimation. The performances of the personalized model and the generalized model were also compared. Results: A mean F1 score of 79.48% was achieved using Convolutional LSTM based on the personalized model. Conclusion: This study demonstrates the potential for the estimation of cold pain intensity from facial expression analysis and shows that the personalized spatial-temporal framework has better performance in cold pain intensity estimation. Significance: This cold pain intensity estimator could allow convenient, automatic, and real-time use to provide continuous objective pain intensity estimations of subjects and patients.

## I. Introduction

Pain is an unpleasant sensory and emotional experience due to actual or potential tissue damage or injury [1]. Pain management and assessment are of importance in health and patient care. Traditionally, pain is measured by patients’ self-reported information. The three most common measurements of self-reported assessment are visual analog scales (VAS), numerical rating scales (NRS), and verbal rating scales (VRS) [2]. Although self-reported assessment is considered as a gold standard to provide important clinical information and help physicians to determine proper treatment for patients, it does have limitations in specific situations. For example, individuals may have limited abilities to verbally tell physicians their pain intensity, such as infants, children, or patients with certain neurological impairments, dementia, disorders of consciousness [3]. Furthermore, some patients who are addicted to drugs may provide higher pain intensity on purpose to obtain excess medication. More important, pain intensity based on patients’ verbal response may be inadequate or delayed which may lead to misdiagnosis and increase medical risks [4], [5]. So there is an increasing demand for automatic, and real-time pain intensity assessment.

Cold pain, also known as cold pressor test (CPT), was first introduced by Hines Jr [6]. It is a cardiovascular test that requires the subjects to put one hand into cold water. The main advantage of the cold pain test could be the convenience [7]. Nowadays, it has become a common pain test used in the clinical settings as well as lab settings.

Over the past few years, researchers have taken advantage of neuroimaging technology for pain intensity assessment which includes functional magnetic tomography imaging (fMRI) [8], positron emission tomography (PET) [9], pupillary diameter [10], and single-photon emission tomography (SPET) [11]. Meanwhile, electroencephalography (EEG) becomes another good indicator of pain intensity, which has shown promising results for evaluating pain intensity by M. Yu *et al.* [12]. The main limitation of EEG is that setting up EEG equipment is time-consuming. Patients may feel uncomfortable and reject the EEG equipment since the EEG cap with gel will be placed on their heads. Lin *et al.* [13], Wang *et al.* [14] also investigated other physiological signals on pain measurement and showed the feasibility of fusing with EEG signals to assess pain. Although significant results were achieved, the drawbacks of these methods are their expensive cost and inconvenient application.

To meet the goal of convenient, automatic, and real-time pain intensity assessment, research in computer vision has become an important part of pain detection, since it goes directly toward an automatic detector of spontaneous facial expressions [15], [16] [17], [18]. Visual painful facial expression can provide the intensity of pain in the face assessed by the Facial Action Coding System (FACS) [19], by which movements of facial muscles with different intensity are coded. Currently, there are two public visual databases focusing on pain. UNBC-Macmaster database [20] consists of 31571 frames from 25 subjects who are suffering shoulder pain, with pain intensity from 0-16 PSPI [21] and 0-10 VAS. BioVid database [22] consists of 17300 frames from 90 subjects who are suffering from stimulated heat pain, with pain intensity from 1-4. These datasets have been used to train models for pain intensity estimation based on facial expression, but they are very challenging to distinguish whether a subject is in pain or not in some cases.

Various algorithms were reported to be useful for facial expression feature extraction. Previous work showed that Active Appearance Models (AAMs) [23] satisfied performance in analyzing spontaneous pain expression. Ashraf *et al.* [17] used landmark features extracted by AAMs and Support Vector Machines (SVMs) as classifiers to predict painful action units for the presence of pain. Hammal and Cohn [24] used the canonical normalized appearance of the face extracted by AAM and 4 separately trained SVMs to classify four levels of pain intensity. Recently, using deep learning frameworks such as Convolutional Neural Networks (CNN) and Recurrent Neural Networks (RNN) has become a trend to deal with emotion estimation as well as pain intensity estimation. There are two kinds of information when employing a deep learning framework for pain intensity estimation: 1. Spatial information, 2. Temporal information. Spatial information, which contains pain-related information, is extracted from every single frame. Although it can exhibit static features, the information between pain expressions, also a key to pain intensity estimation, will be lost. Temporal information, on the other hand, can describe relevant dynamic information among consecutive frames. Jiang *et al.* [25] proposed a novel deep neural network framework to assess major depressive disorder. Zhou *et al.* [26] proposed an end-to-end pain intensity regression framework based on AAM-warped facial images and recurrent convolutional neural networks to predict pain intensity. Rodriguez *et al.* [15] proposed a combined CNN with Long Short-Term Memory networks (LSTM) framework. The LSTM was linked to the top of a VGG-16 [27], in which raw images were used instead of facial landmarks as the input of the CNNs and features from the fc6 layer were used to feed the LSTM. The top layer of LSTM was found to improve the results significantly. Convolutional LSTM (C-LSTM) was introduced by Shi *et al.* [28], in which fully connected LSTM was extended by convolutions. C-LSTM is suitable for spatial-temporal data due to the advantage of its inherent convolutional structure.

In this paper, we investigate the plausibility of using three deep learning architectures, Inception V3, VGG-LSTM, and C-LSTM to automatically estimate cold pain intensity in videos based on facial expressions. To the best of our knowledge, this is the first work using facial expressions to estimate cold pain intensity. The architecture will learn an end-to-end pattern without the help of intermediate representations such as the FACS. Two models, personalized and generalized models, are also developed to investigate the performance on the cold pain intensity estimation task. The rest of the paper is organized as follows. Section II describes the process of cold pain intensity based on facial expression experimental design and dataset establishment. Section III contains the proposed architecture and models. Section IV and section V provide the obtained results and discussions. Section VI contains limitations and future work. Section VII is the conclusion.

## II. Experiment

### A. Participants

Twenty-nine subjects, aged 19-22 and from Northeastern University, were recruited to take part in this experiment. We included 18 females and 11 males since gender differences might have effects on the tolerance of the pain. All subjects were right-handed and healthy. Prior to the experiment, the detailed experimental procedure, participants’ role, and other related information were provided to the subject in a written consent form and by oral explanation from the experimenters.

### B. Task

**FIGURE 1.**
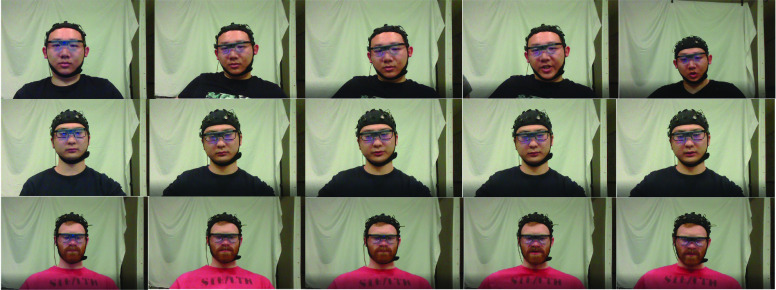
Example facial expression data from the cold pain dataset.

The experiment was to investigate facial expressions and some physiological signals (e.g., EEG signals, eye movement, etc.) in different pain levels. In this paper, we only focused on the facial expressions. Cold pain was selected as the stimuli of the pain in this experiment. Subjects were asked to put their right hands into the iced water so that the pain was induced. Every subject was asked to participate 3 times on 3 different days during the week. The whole task was completed within 7 days. When participating in the experiment, subjects were asked to show their natural facial expressions when they felt pain. The image data were captured and stored by a GoPro 5. Fig. 1 shows examples of facial expressions captured during the cold pain experiment.

### C. Procedure

**FIGURE 2.**
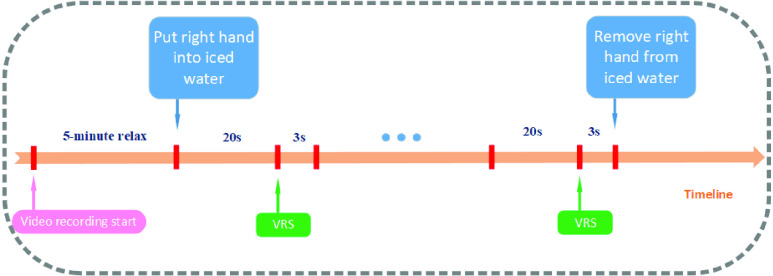
Illustration of the cold pain experiment procedure.

The detailed procedure of the experiment is presented as follows. Each subject with eyes open was asked to sit in a chair at a distance of 1m from a computer screen. The diagram of the experimental procedure is shown in Fig. 2. The “$\ldots $” symbol means that the subject was asked to report his/her pain intensity multiple times (every 20 seconds).

**FIGURE 3.**
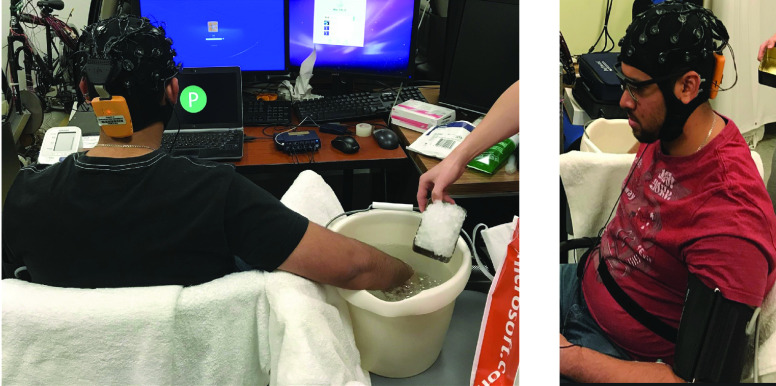
Cold pain experiment where the subject’s right hand was in the iced water.

Firstly, each subject was given a 5-minute relaxation time. Then the subject was instructed by the computer screen to put his/her right hand into a barrel with iced water. Fig. 3 shows the scenario when a subject was doing the cold pain experiment.

**FIGURE 4.**
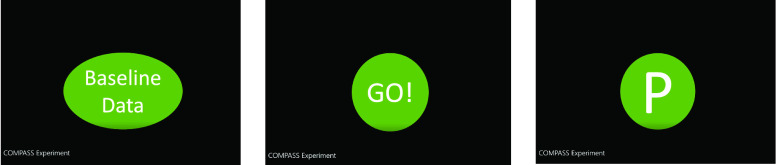
The process of the instruction video shown to the subjects during the cold pain experiment. The first picture shows up when the video recording starts. The subjects are asked to put their right hands into the iced water when “GO” shows up. The subjects are asked to report pain intensities when the “P” shows up.

During the experiments, the subjects were required to remain as still as possible and face the camera directly. The camera was placed on the top of the screen. When the experiment started, an instruction video was shown to the subjects. In the first 5 minutes, the video showed ‘Baseline data’ which meant no-pain data was recorded. Then the video showed ‘Go!’ which meant the subject should put his or her right hand into the iced water. After that, subjects were asked to report their pain intensity from 0–10 based on numerical rating scales (NRS, 0: no pain, 1: barely noticeable pain, 5: mild pain, and 10: worst pain) every 20 seconds. At the end every 20 seconds, the video showed ‘P’ which meant the subject needed to report his or her pain level. Fig. 4 shows the process of the instruction video shown to the subjects during the cold pain experiment.

**FIGURE 5.**
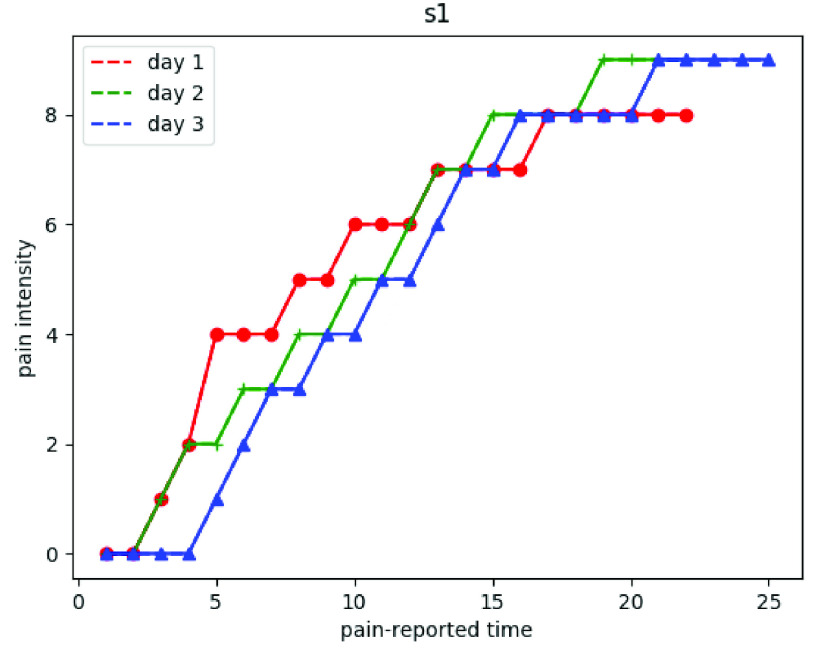
The trend of pain intensity of a randomly selected subject.

Fig. 5 shows the true pain intensities from one randomly selected subject among the total 29 subjects based on his self-reporting. The red, green, and blue lines demonstrate his pain intensities on day 1, day 2, and day 3, respectively. The X-axis corresponds to the pain reported time and the Y-axis corresponds to the pain intensity from 0 to 10. From the figure we can see that the pain intensity is increasing as time goes on.

To simplify the estimation task, we only use three pain intensities: no pain (0), mild pain (1-5), and severe pain (6-10). Image data were recorded when the experiment started. The obtained data were divided into training, validating, and testing sets for proposed architecture. The whole experimental procedure would stop once the subject was not able to bear the pain. All subjects were from Northeastern University and the procedure was approved by the Northeastern University Institutional Review Board (IRB #17-01-25).

### D. Preprocessing

**FIGURE 6.**
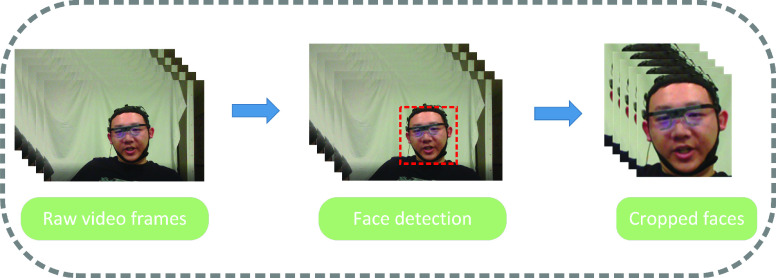
Preprocessing on the raw video frames.

The original video frames contain a large portion of the subject’s body. Since we just want to focus on the facial expressions, face detection is applied to obtain the cropped face which is further used as the input of the model. Fig. 6 shows the preprocessing pipeline. Since we only investigate three pain intensities, the facial expression data under different pain intensities were collected according to the timeline.

## III. Methods

### A. Architectures

We investigated three main deep learning architectures: deep CNN InceptionV3 [29] where single-frame was taken as input, the CNN+LSTM architecture [15] where VGG-16 was the CNN that extracted spatial information and LSTM was linked to exploiting the temporal information, and the fully recurrent C-LSTM. The main advantage of C-LSTM is that spatial and temporal information can be extracted at the same time.

#### 1) Inception V3

Inception V3 is a convolutional neural network. It is made up of symmetric and asymmetric building blocks, which contain convolutions, average pooling, max pooling, concats, dropouts, and fully connected layer. In this paper, this architecture is trained with RMSProp as the optimizer.

#### 2) VGG-LSTM

We investigated the performance of the hybrid VGG-LSTM on our cold pain facial expression dataset. VGG-Faces [30] was used to learn the spatial information of facial features. Then the LSTM was linked to exploit the temporal information between the frames. We fine-tuned VGG-Faces and the last layer was replaced by a fully connected layer with three pain intensities for estimation. The fully connected layer was randomly initialized. After fine-tuning, the features of the output of the VGG were extracted and set as the input of the LSTM. The initial learning rate was set to 0.001, dropout with probability was set to 0.2, and ADAM was chosen as the optimizer to overcome the hyper-parameter tuning problem. Ten frames were extracted when training the LSTM. Data augmentation including horizontal flipping, random cropping, and shading by adding Gaussian noise was implemented.

#### 3) Convolutional LSTM (C-LSTM)

C-LSTM was introduced by Shi *et al.* [28]. Fully connected LSTM (FC-LSTM) was extended by convolutions in both the input-to-state and state-to-state transitions. This enables C-LSTM to preserve both parameter sharing and location invariance from convolutional layers and maintain the recurrent settings at the same time. The fully connected LSTM structure contains too many redundant connections and can hardly extract the local consistencies. For example, the input to the LSTM must be flattened to a 1-D vector which will lead to the loss of spatial grid patterns of images. In addition, although LSTM is able to extract both spatial and temporal information, the two kinds of information are captured separately, which may result in loss of important spatial-temporal information. C-LSTM is able to overcome these drawbacks by extracting both spatial and temporal features simultaneously. The key equation of a C-LSTM unit can be interpreted as follows:

\begin{align*} i(t)=&\sigma (W_{xi} * x(t)\!+\! W_{hi} * h(t\!-\!1)\!+ \!W_{hi} \circ c(t-1)+b_{i}) \\ \tag{1}\\ f(t)=&\sigma (W_{xf} * x(t)\!+ \!W_{hf} * h(t\!-\!1)\!+ \!W_{hf} \circ c(t-1)+b_{f}) \\ \tag{2}\\ z(t)=&\tanh (W_{xc} * x (t)\,\,+ W_{hc} * h(t-1) + b_{c}) \tag{3}\\ c(t)=&f(t) \circ c(t-1) + i(t) \circ z (t) \tag{4}\\ o(t)=&\sigma (W_{xo} * x(t)\!+ \!W_{ho} * h(t\!-\!1)\!+ \!W_{co} \circ c(t)+b_{o}) \tag{5}\\ c(t)=&o(t) \circ \tanh (c(t)) \tag{6}\end{align*}

where * and $\circ $ denote the convolution and Hadamard product, respectively. The sequences consisting of 10 frames are trained and extracted without overlap. The labels are also predicted sequence-wise so that the information we get will contain the past frames. The learning rate and dropout probability are set to 0.001 and 0.2, respectively. Early stopping of 20 epochs is also applied to overcome the overfitting problem. A max pooling and a batch normalization layers are between each stacked layer.

### B. Models

#### 1) Personalized Models

**FIGURE 7.**
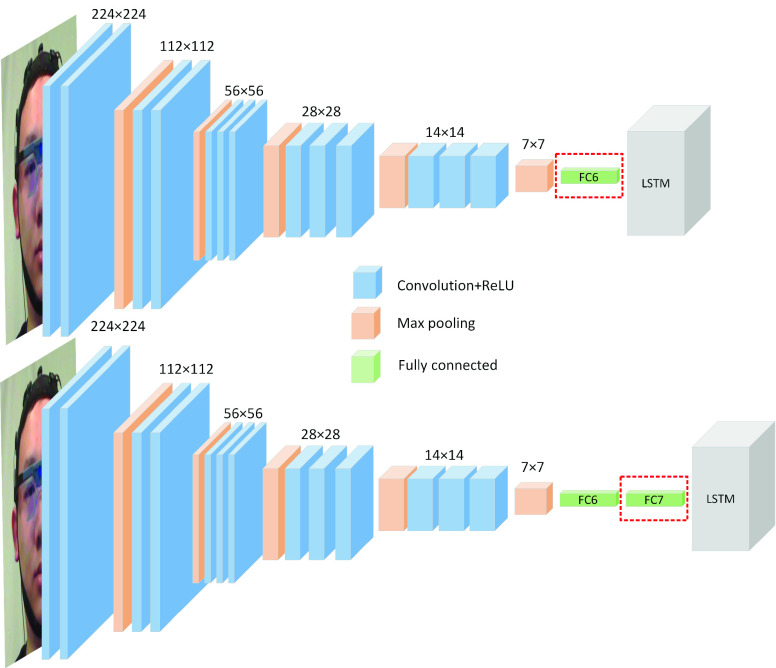
The two proposed VGG-LSTM structures.

The ground truth for the proposed deep learning architecture is the self-reported pain intensity. Due to the individual differences in pain tolerance, we firstly investigate models based on personalized pain intensity estimation system. These models are to build three architectures (described in Section 3A) based on each individual’s facial expression. As a result, these models will only deal with individual information and neglect the effect of individual differences. There is 80% of collected facial expression data from each subject that is used to train the personalized Inception V3, VGG-LSTM, and C-LSTM architecture. The rest data are separated into validation set and testing set equally. The validation set is used to prevent the models from overfitting and the testing set is used to measure the accuracy of the models. To find the optimal architecture of models for the pain intensity estimation, we investigate the variability for the VGG-LSTM and C-LSTM. In the VGG-LSTM model, two candidates are proposed. The first candidate is VGG-LSTM-1, in which the input features for the LSTM are extracted from fc6 layer of the VGG. The second candidate is VGG-LSTM-2, in which the input features for the LSTM are extracted from the fc7 layer of the VGG. The architectures are shown in Fig. 7.

**FIGURE 8.**
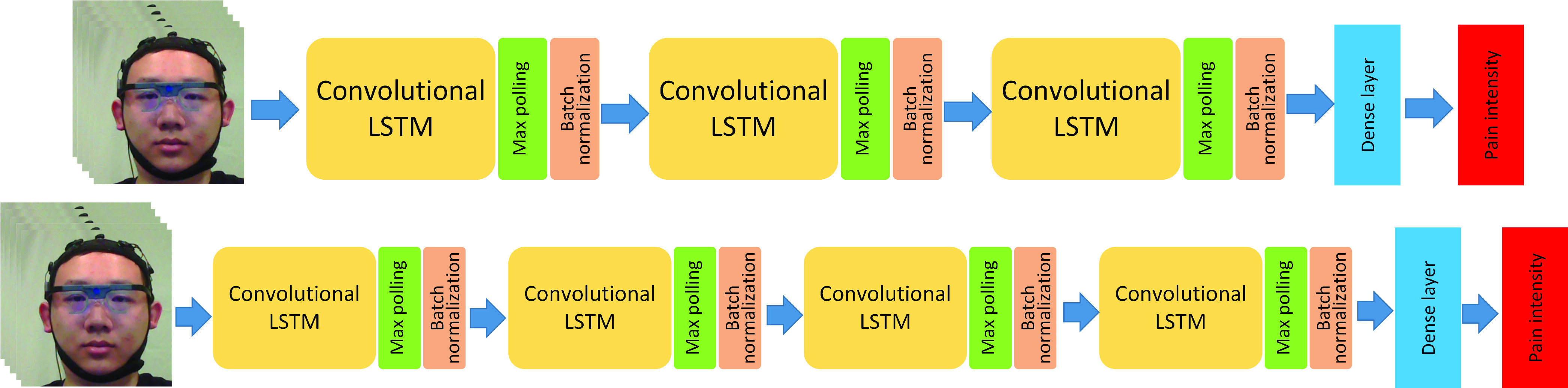
The two proposed C-LSTM structures, one is with three stacked layers and the other is with four stacked layers.

In the C-LSTM architecture, two types of candidates are proposed. The first candidate, called C-LSTM-1, contains three stacked layers and the second candidate, C-LSTM-2, contains four stacked layers. The architectures of the two C-LSTM are shown in Fig. 8.

#### 2) Generalized Models

To further investigate the possibility of the generalized models, we use two strategies for training and testing. The first strategy is to use 80% of all the subjects’ facial expression data for training and 10% of all data for testing. The remaining 10% is used as validation set to overcome the overfitting problem. The second strategy is the 5-fold cross validation strategy. Since the dataset consists of 29 subjects, we divide them into 5 disjoint sets and run 5-fold cross validation. Four sets contain 24 subjects for training and 5 subjects for testing. The remaining one contains 25 subjects for training and 4 subjects for testing.

## IV. Experimental Results

This section provides the experimental results obtained from the proposed architectures in two models using the dataset of facial expression-based cold pain intensity estimation.

Section IV A introduces the strategy. Section IV B gives the results of performance on the proposed models with all the subjects during three days’ experimental data.

### A. Evaluation Method

As mentioned in Section II, our cold pain intensity evaluation is a three-class task of no pain (NP), mild pain (MP), and severe pain (SP). The performance is evaluated by using an error matrix, which is a table where each row and each column represent an actual class and a predicted class, respectively. Based on the statistical information of the confusion matrix, the average testing accuracy, precision, specificity, sensitivity, and F1 score are calculated as the performance evaluation metrics in our paper. The reason why the F1 score is used is that it is a more cautious measure in a situation when classes are imbalanced [31]. The confusion matrix with three classes is shown in Table 1, where NP, MP, and SP represent no pain, mild pain, and severe pain, respectively.

**TABLE 1:** Confusion Matrix for Three-Class Classification

		Predicted pain intensity		
		NP	MP	SP
True pain intensity	NP	$x_{11}$	$x_{12}$	$x_{13}$
	MP	$x_{21}$	$x_{22}$	$x_{23}$
	SP	$x_{31}$	$x_{32}$	$x_{33}$

Firstly, three one-vs-all confusion matrices for each class $C_{i}(i=1,2,3)$ are calculated. Secondly, four factors $TP_{i}$, $TN_{i}$, $FP_{i}$, $FN_{i}$ are defined. Then the $Precision_{i}$, $Specificity_{i}$, $Sensitivity_{i}$ and $F1_{i} Score$ for $C_{i} $ and accuracy can be calculated as follows:

\begin{align*} Precision_{i}=&\frac {TP_{i}}{TP_{i}+FP_{i}} \tag{7}\\ Specificity_{i}=&\frac {TN_{i}}{TN_{i}+FP_{i}} \tag{8}\\ Sensitivity_{i}=&\frac {TP_{i}}{TP_{i}+FN_{i}} \tag{9}\\ F1_{i}=&\frac {2}{Sensitivity_{i}^{-1}+Precision_{i}^{-1}} \tag{10}\\ Accuracy=&\frac {\sum _{i=1}^{3}TP_{i}}{N} \tag{11}\end{align*}

where $TP_{i}=x_{ii}$ denotes the total number of true-positive cases for $C_{i} $, $TN_{i}=\sum _{j=1,j\neq i}^{3}\sum _{k=1,k\neq i}^{3}x_{jk}$ denotes the total number of true-negative cases for $C_{i} $, $FP_{i} = \sum _{j=1,j\neq i}^{3} x_{ji} $ denotes the total number of false-positive cases for $C_{i} $, and $FN_{i} = \sum _{j=1,j\neq i}^{3} x_{ij} $ denotes the total number of false-negative cases for $C_{i} $, $N$ is the total number of samples for each test.

### B. Results

In our study, two models, the personalized model and the generalized model, are investigated. In each model, three proposed architectures are tested using the dataset of facial expression-based cold pain intensity estimation. The details of the proposed architectures are shown in Table 2.

**TABLE 2:** Overview of Details for the Three Evaluated Models

Model	Input data	Batch size	Sequential	Optimizer
Inception V3	[299, 299, 3]	100	No	RMSProp
VGG-LSTM	[10, 224, 224, 3]	16	Yes	ADAM
C-LSTM	[10, 128, 128, 3]	16	Yes	Adadelta

**FIGURE 9.**
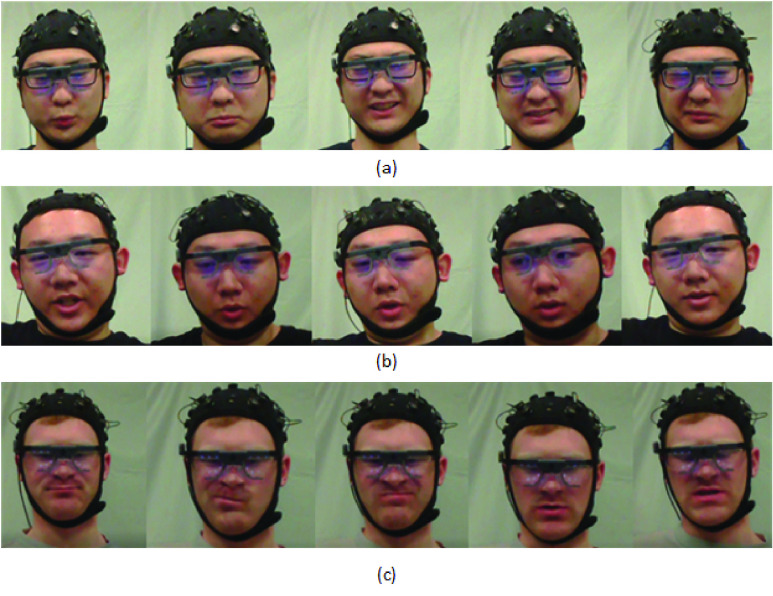
Success and failure samples for the proposed models. (a): Samples successfully classified by the three architectures. They belong to the pain sequences. (b): Samples successfully classified by the VGG-LSTM and C-LSTM, misclassified by the Inception V3. These samples are captured when the subject is reporting his pain intensity. (c): Samples misclassified by the three architectures. These samples are captured during the baseline time.

**FIGURE 10.**
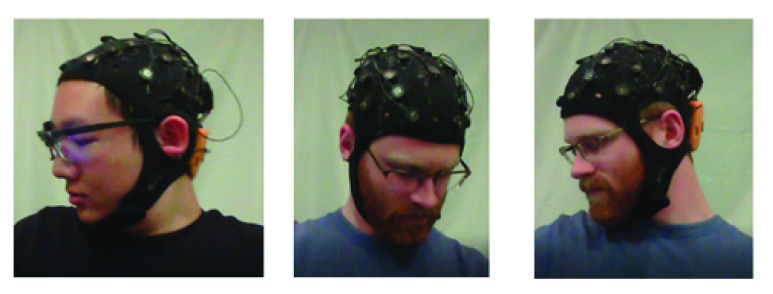
Samples that are not detected by the face detector will not be used to train models.

First, performances of the three architectures under personalized model are compared. Table 3 shows the results on three architectures of the personalized model in three pain intensities. The mean F1 Scores for Inception V3, VGG-LSTM-1, VGG-LSTM-2, C-LSTM-1 and C-LSTM-2 are 65.17%, 72.03%, 79.46%, 76.69% and 79.48%. The F1 Score of Inception V3 is obviously lower than the VGG-LSTM and C-LSTM, which means that the temporal information is important for continuous pain intensity estimation. The F1 score of VGG-LSTM-1 is lower than VGG-LSTM-2, which shows that selecting the features from fc7 layer as the input of LSTM performs better than that from fc6 layer. The F1 score of C-LSTM-2 is higher than the F1 score of C-LSTM-1, which shows that the C-LSTM with 4 stacks is better than that with 3 stacks in our pain intensity estimation task. We also notice that most of the NP F1 Score achieves the highest among the three pain intensities. The reason is that the data of NP is much more than the other two intensities, demonstrating that the deep learning architecture needs more data to train. And the MP F1 Score achieves the lowest. Besides the amount of the data, another reason may be that the difference between the MP and SP is not that obvious. It is even hard for people to judge some images between MP and SP. Fig. 9 shows exemplary success and failure cases for the three proposed architectures. Fig. 9.(a) are samples that are successfully classified by the three architectures. They belong to the pain sequences. Fig. 9.(b) are samples that are successfully classified by the VGG-LSTM and C-LSTM but misclassified by the Inception V3. They are captured when the subject was reporting the pain intensity. Due to the lost of temporal information, Inception V3 model misclassified them as the pain sequence. Fig. 9.(c) are samples that are misclassified by all three architectures. These are captured during the baseline time when the subject hasn’t put his hand into the iced water. He was relaxing himself and was not feeling pain. All three architectures misclassified them as the pain sequence. Fig. 10 shows samples that are not detected by the face detector. These samples will not be used to train the models. Before the experiment, we asked the subjects to keep face straightforward and try not to move their heads.

**TABLE 3:** Overview of Details for the Evaluated Personalized Models

Architecture	Pain Intensity	Precision (%)	Specificity (%)	Sensitivity (%)	F1 Score (%)
Inception V3	NP	62.85	79.86	67.38	65.04
	MP	67.64	81.62	60.24	63.73
	SP	65.96	80.84	67.53	66.74
VGG-LSTM-1	NP	72.82	83.64	78.41	75.51
	MP	68.72	83.56	63.54	66.03
	SP	73.71	85.48	70.12	71.87
VGG-LSTM-2	NP	77.84	87.95	84.08	80.84
	MP	76.12	87.79	78.14	77.12
	SP	83.88	91.14	77.23	80.42
C-LSTM-1	NP	78.96	83.59	76.92	77.93
	MP	75.06	56.74	78.52	76.75
	SP	72.38	83.84	78.65	75.39
C-LSTM-2	NP	78.36	86.52	81.75	80.02
	MP	76.16	86.08	79.46	77.78
	SP	83.64	89.41	77.84	80.64

We use two strategies for the generalized model. In strategy 1, 80% of all the subjects’ facial expression data is for training and 10% of all data for testing. The remaining 10% is used as the validation set to overcome the overfitting problem. In strategy 2, 5-fold cross validation strategy is used to estimate the performance of the proposed architecture. Table 4 shows the results of the proposed architecture on the generalized model. The performance of Inception V3 achieves the worst among all the architecture, which continues to show that satisfactory results cannot be obtained if temporal information is not taken into account. The C-LSTM-2 achieves the best, followed by the VGG-LSTM-2. However, if compared with the personalized model, all architectures of the generalized model are worse than those of the personalized model.

**TABLE 4:** Overview of Details for the Evaluated Generalized Models

Model	Strategy 1 (F1 Score %)	Strategy 2 (F1 Score %)
Inception V3	53.51	49.23
VGG-LSTM-1	59.32	61.82
VGG-LSTM-2	63.67	65.86
C-LSTM-1	62.75	61.27
C-LSTM-2	69.58	68.73

## V. Discussion

In this study, a cold pain experiment was designed to collect facial expression video data when subjects put their hands in iced water which acted as the pain inducer. After the data acquisition and preprocessing, we implemented deep learning frameworks to estimate the pain intensities based on the facial expression data. The study discovered three findings that greatly contribute to cold pain research. First, the pain intensities kept going up as time goes when the subjects put their hand in the iced water. From Fig. 5 we can see that the pain intensities begin at pain intensity 0 and end at a very high level. Second, the personalized model achieved better performance than the generalized model. Unlike the general emotion recognition task, pain related facial expressions are hard to distinguish and are more dependent on the individuals. In our study, the subjects were asked to show their natural expression when they feel pain. However, expressions varied between different subjects, even under the same pain intensities. Moreover, even for the same subject, expressions varied between the different days. Based on these factors, a more personalized model should be more useful than a generalized model. Third, the temporal information was important for our continuous cold pain intensity estimation task. In both personalized and generalized models, we investigated the performance of three deep learning architectures, Inception V3, VGG-LSTM, and C-LSTM. Moreover, we also developed two structures for both VGG-LSTM and C-LSTM. The results showed that both VGG-LSTM and C-LSTM have more promising results than Inception V3, demonstrating a positive effect of temporal information on the final pain intensity estimation. In addition, the two structures with more layers and stacks of VGG-LSTM and C-LSTM performed better than those with simpler structures, which showed that they could extract more information from the image data.

## VI. Limitations and Future Work

This study discovered several features that can serve as building blocks for future cold pain research. Several limitations are as follows. First, we only used facial expressions as the indicator to estimate the pain intensities. Since we believe that the fusion of the physiological signals with facial expression analysis will boost the final pain intensity estimation performance, future research will investigate the fusion strategy. Second, the ground truth in our study was based on subjects’ self-reported pain intensities. Some research [20] used professional pain observers to measure the pain intensities based on the facial movements. In future research, pain observers will be trained in our study to give a more reliable ground truth. Third, participants in our experiment were all health subjects in the university. Considering more data needed for training the deep learning model, we will recruit real patients in the future and collect more data to enhance the robustness of our system.

## VII. Conclusion

Pain assessment plays a key role in health care. Facial expression-based pain intensity estimation is becoming more important due to its advantages of convenience. This paper presented a facial expression database for cold pain intensity estimation. The database contained facial express and subjective report data from 29 subjects under three levels of cold pain. We investigated three deep learning architectures to assess cold pain intensity. We next investigated two models, the personalized model and the generalized model, using our database. We demonstrated that facial expression data can be used as an objective indicator of cold pain. We further demonstrated that the architectures with spatial-temporal information performed better than the architecture with only spatial information. Finally, our models also showed that the personalized model may serve better than the generalized model as the cold pain intensity estimator.
